# Circulating cell-free DNA and tumor DNA dynamics in obstructive colon cancer undergoing neoadjuvant chemotherapy following stent placement

**DOI:** 10.3389/fsurg.2025.1581993

**Published:** 2025-07-16

**Authors:** Kunning Zhang, Jiagang Han, Zhiwei Zhai

**Affiliations:** ^1^ Department of Pathology, Beijing Chao-Yang Hospital, Capital Medical University, Beijing, China; ^2^ Department of General Surgery, Beijing Chao-Yang Hospital, Capital Medical University, Beijing, China

**Keywords:** circulating cell-free DNA, circulating tumor DNA, obstructive colon cancer, self-expanding metallic stent (SEMS), neoadjuvant chemotherapy

## Abstract

**Background:**

To improve the prognosis of patients with obstructive colon cancer, performing neoadjuvant chemotherapy after self-expanding metallic stent (SEMS) placement followed by elective surgery is currently one of the treatment methods for obstructive colon cancer. However, the oncological risks of this treatment approach are currently unclear. To evaluate the oncological risks of this treatment model by detecting changes in circulating cell-free DNA (cfDNA) and circulating tumor DNA (ctDNA) during the stent placement combined with neoadjuvant chemotherapy process.

**Methods:**

From January to December 2023, 10 patients with obstructive colon cancer who received neoadjuvant chemotherapy after SEMS placement, followed by surgical treatment, were included in this study. Blood samples were collected one day before stent placement, 3 days after stent placement, one day before surgery, and one day after surgery. cfDNA and ctDNA in the blood were detected and analyzed.

**Results:**

The stent placement success rate was 100%, with no cases of perforation, displacement, or re-obstruction, and no perioperative deaths. After neoadjuvant chemotherapy, peripheral ctDNA decreased compared to before stent placement. There were no statistically significant differences in cfDNA and ctDNA changes at the four time points during the treatment process.

**Conclusions:**

This study did not find an increase in ctDNA after stent placement combined with chemotherapy, suggesting that the model of stent placement combined with neoadjuvant chemotherapy for obstructive colon cancer may be a safe and reliable therapy.

## 1 Introduction

Bowel obstruction is one of the most common reasons for emergency surgery in colorectal cancer. However, the complication and mortality rates of emergency surgeries are significantly higher than those of elective surgeries (1). Compared to emergency surgery, stent placement as a transitional treatment before surgical intervention provides a time window to improve the patient's overall condition, allowing for a comprehensive assessment of the disease. This approach increases the laparoscopic surgery rate, improves the primary anastomosis rate, reduces the stoma creation rate, decreases the risk of wound infections, and lowers surgical mortality rates (2–4).

Although stent placement is becoming a more common treatment method for obstructive left-sided colon cancer, there is still debate about its long-term impact on patient prognosis (5). A meta-analysis by Foo et al. (6) revealed that, compared to emergency surgery, patients undergoing stent placement had worse survival outcomes and higher local recurrence rates. However, some recent studies indicate no significant difference in long-term survival between patients undergoing stent placement and those receiving emergency surgery (7, 8). Currently, no reliable randomized controlled trial data exists to evaluate this issue.

Maruthachalam et al. (9) previously reported that stent placement induced the shedding of circulating tumor cells (or their fragments) into the bloodstream in 8 out of 20 patients, as detected using cytokeratin 20 messenger RNA (mRNA). Yamashita et al. (10) observed elevated circulating tumor cells in peripheral blood following stent placement, with early postoperative metastasis leading to death in patients with elevated circulating tumor cells. Recent studies have found that peripheral ctDNA levels significantly increased on day 3 after stent placement for bowel obstruction, and cfDNA levels were markedly elevated on day 7 post-stent placement. These factors may contribute to poor patient prognosis (11). Multiple studies have confirmed that postoperative ctDNA positivity is associated with an increased risk of recurrence. ctDNA can serve as a prognostic biomarker and an early, real-time marker for assessing the benefits of adjuvant therapy. Continuous dynamic ctDNA monitoring can also be used for molecular relapse surveillance (12).

Neoadjuvant chemotherapy enhances the likelihood of achieving negative surgical margins, treats potential lymph node and/or distant micrometastases at an early stage, and provides an opportunity to downstage colorectal cancer (13). Neoadjuvant chemotherapy can also increase resectability in cases of acute left-sided malignant colorectal obstruction (14). Recent studies have investigated the use of neoadjuvant chemotherapy during the interval between stent placement and surgery to improve long-term patient outcomes (15–17). Our center previously reported that administering 2–3 cycles of chemotherapy during the interval between stent placement and surgery did not increase surgical complication rates and was well-tolerated by patients (16). Zhang et al. compared neoadjuvant chemotherapy following stent placement with surgery alone and found that the neoadjuvant group had lower stoma creation rates, fewer postoperative complications, shorter postoperative bowel function recovery times, reduced ICU stays, and shorter overall hospital stays. One-year follow-up showed no difference in recurrence-free survival (RFS) between the two groups; however, long-term outcomes still require further research (17).

During SEMS placement followed by neoadjuvant chemotherapy and scheduled surgery for treatment of obstructing colonic cancer, the changes of circulating cfDNA and ctDNA have not been reported. In this prospective study we analyze the changes of circulating cfDNA and ctDNA to explore the oncologic risk for SEMS follow by neoadjuvant chemotherapy.

## 2 Materials and methods

### 2.1 Patients

This prospective, observational study was approved by the Ethics Committee of Beijing Chaoyang Hospital, Capital Medical University and performed in accordance with principles within the Helsinki Declaration. Informed consent was obtained from all patients before treatment.

In 2023, patients diagnosed with primary obstructive colorectal cancer at the Department of General Surgery, Beijing Chaoyang Hospital, Capital Medical University, were enrolled in this study based on inclusion and exclusion criteria.

Inclusion criteria:

1. Clinically and radiologically diagnosed with complete colorectal obstruction.
2. Pathologically confirmed primary colonic adenocarcinoma.

Exclusion criteria:

1. Patients who underwent emergency surgery without opting for self-expanding metallic stent (SEMS) placement.
2. Patients presenting with severe abdominal pain and distension, along with peritonitis at the time of consultation.
3. Patients unwilling to accept the study protocol or sign the informed consent form.

### 2.2 Treatments

Clinically, bowel obstruction is defined as the complete inability of the intestine to pass stool and gas. Acute left-sided colonic obstruction can be diagnosed through clinical symptoms (abdominal distension, pain, inability to defecate or pass gas), physical examination, abdominal x-rays, and abdominal computed tomography (CT). Once the diagnosis is confirmed at the time of consultation, an experienced gastroenterologist immediately performs a colonic stent placement under endoscopy and conducts tissue biopsy [SEMS (WallFlex; Boston Scientific Corporation, Natick, MA, USA)]. Abdominal CT is used to confirm the degree of stent expansion and its position. Technical success is defined as the successful placement of the stent at the site of narrowing. Clinical success is defined as satisfactory bowel decompression within 24 h after stent placement, with alleviation of the patient's clinical obstructive symptoms.

One week after stent placement, patients begin two cycles of chemotherapy under the CAPOX regimen. This consists of oxaliplatin 130 mg/m² administered intravenously on day 1, and capecitabine 1,000 mg/m² taken orally twice daily from days 1–14, repeated every three weeks. Toxicity is assessed according to the National Cancer Institute Common Toxicity Criteria (NCI-CTC, version 4.0). Two weeks after completing chemotherapy, patients undergo elective surgery. The choice between open surgery and laparoscopic surgery is determined by the hospital and the attending physician.

### 2.3 Blood collection and DNA isolation and capture-based targeted DNA sequencing

Blood samples were collected at four distinct time points: 1 day prior to SEMS implantation, 3 days post-SEMS, 1 day before surgery, and 3 days following surgery. These four points represent the key aspects that patients must meet to undergo the treatment model. If an adverse event occurs at any of these stages, the patient may no longer be eligible for treatment according to our proposed protocol. Ten milliliters of blood were collected in BD Vacutainer plasma preparation tubes (Becton, Dickinson and Company, Franklin Lakes, NJ, USA). Plasma samples were prepared by centrifugation at 1,900×g for 10 min at 4℃ within 2 h after blood collection. DNA isolation and targeted sequencing were performed in Burning Rock Biotech, a commercial clinical laboratory accredited by the College of American Pathologists (CAP) and certified by the Clinical Laboratory Improvement Amendments (CLIA), according to optimized protocols as described previously (18, 19). Briefly, circulating cell-free DNA (cfDNA) was extracted from 4 to 5 ml of plasma samples using a QIAamp Circulating Nucleic Acid kit, according to the manufacturer's standard protocol (Qiagen, Hilden, Germany). The extracted cfDNA was quantified by Qubit dsDNA HS assay (Thermo Fisher Scientific, Waltham, MA, US). Germline DNA was isolated from matched peripheral blood leucocytes that were separated by Ficoll gradient density centrifugation. Fragments between 200 and 400 bp from cfDNA were purified (Agencourt AMPure XP Kit, Beckman Coulter, CA, USA), hybridized with capture probe baits, selected with magnetic beads, and amplified. Target capture was performed using a commercial panel consisting of 168 cancer-related genes. The fragments' quality and size were assessed by high sensitivity DNA kit using Bioanalyzer 2100 (Agilent Technologies, CA, USA). Indexed samples were sequenced on Nextseq 500 (Illumina, Inc., CA, USA) with paired-end reads and an average sequencing depth of 10,000×.

### 2.4 Sequence data analysis

Sequence data were mapped to the reference human genome (hg19) using Burrows-Wheeler Aligner version 0.7.10. Local alignment optimization, duplication marking, and variant calling were performed using Genome Analysis Tool Kit version 3.2, and VarScan version 2.4.3. Plasma samples were compared against white blood cell control to identify somatic variants. Variants were filtered using the VarScan fpfilter pipeline, and loci with depths less than 100 were filtered out. Base calling in plasma samples required at least eight supporting reads for single nucleotide variations (SNVs) and two supporting reads for insertion-deletion variations (Indels), respectively. Variants with population frequency over 0.1% in the ExAC, 1,000 Genomes, dbSNP, or ESP6500SI-V2 databases were grouped as single nucleotide polymorphisms (SNPs) and excluded from further analysis. The maxAF is the maximum value of variant allele frequency in the sample. The remaining variants were annotated with ANNOVAR (2016-02-01 release) and SnpEff version 3.6. Structural variations (SVs) were analyzed using Factera version 1.4.3.

Copy number variations (CNVs) were analyzed based on the depth of coverage data of capture intervals. Coverage data were corrected against sequencing bias resulting from GC content and probe design. The average coverage of all captured regions was used to normalize the coverage of different samples to comparable scales. The copy number was calculated based on the ratio between the depth of coverage in tumor samples and the average coverage of an adequate number (*n* > 50) of samples without CNVs as references per capture interval. CNV is called if the coverage data of the gene region was quantitatively and statistically significant from its reference control. The limit of detection for CNVs is 1.5 for copy number deletion and 2.64 for copy number amplifications.

### 2.5 Pathologic examination

The 8th edition of the American Joint Committee on Cancer (AJCC) TNM system was used for staging. The system used to grade tumor response was recommended by the AJCC cancer Staging Manual modified from Ryan R.

### 2.6 Statistical analysis

Descriptive statistics were presented as counts with percentages or medians with interquartile ranges (IQRs). Categorical data were compared using the Chi-squared test and comparisons of continuous data were conducted using the Mann–Whitney test, as appropriate. All analyses were conducted using R version 4.0.2. A *P* < 0.05 for two-tailed tests was considered statistically significant.

## 3 Results

### 3.1 Patient characteristics

From January to December 2023, a total of 16 patients diagnosed with acute obstructive colon cancer were treated in our department. Some patients were excluded for the following reasons: 4 cases underwent emergency surgery, 1 case presented with peritonitis, and 1 case declined to sign the informed consent form. Ultimately, 10 patients were included in the analysis for this study.

The characteristics of the patients are summarized in Table 1. The success rate of stent placement was 100%, with no cases of perforation, displacement, or re-obstruction, and no perioperative mortality. Among the 3 patients with metastases, all had solitary liver metastases, and liver tumor resection was performed concurrently during surgery.

**Table 1:** Clinical characteristics of patients.

Clinical characteristics of patients	Overall
	(*n* = 10)
Age	
Median [IQR]	65.50 [57.75, 73.75]
Sex	
Female	3 (30.0)
Male	7 (70.0)
Tumor site	
Transverse colon	1 (10.0)
Descending colon	2 (20.0)
Sigmoid colon	7 (70.0)
Tumor	
T3	3 (30.0)
T4	7 (70.0)
Node	
N0	1 (10.0)
N1	4 (40.0)
N2	5 (50.0)
Metastases	
M0	7 (70.0)
M1	3 (30.0)
Staging	
III	7 (70.0)
IV	3 (30.0)
Differentiation	
Moderate-low	2 (20.0)
Moderate	5 (50.0)
N/A	3 (30.0)
Abdominal metastasis at diagnosis	
Yes	5 (50.0)
No	5 (50.0)
Abdominal metastasis at diagnosis/during treatment	
Yes	8 (80.0)
No	2 (20.0)
Vascular invasion	
Yes	4 (40.0)
No	6 (60.0)
Perineural invasion	
Yes	2 (20.0)
No	8 (80.0)
TRG	
1	2 (20.0)
2	1 (10.0)
3	7 (70.0)

Data are expressed as number (percent) or median [IQR].

IQR, interquartile range; N/A, not available; TRG, tumor regression grading.

### 3.2 Variation analysis of cfDNA identified by target-sequencing

**Figure 1:**
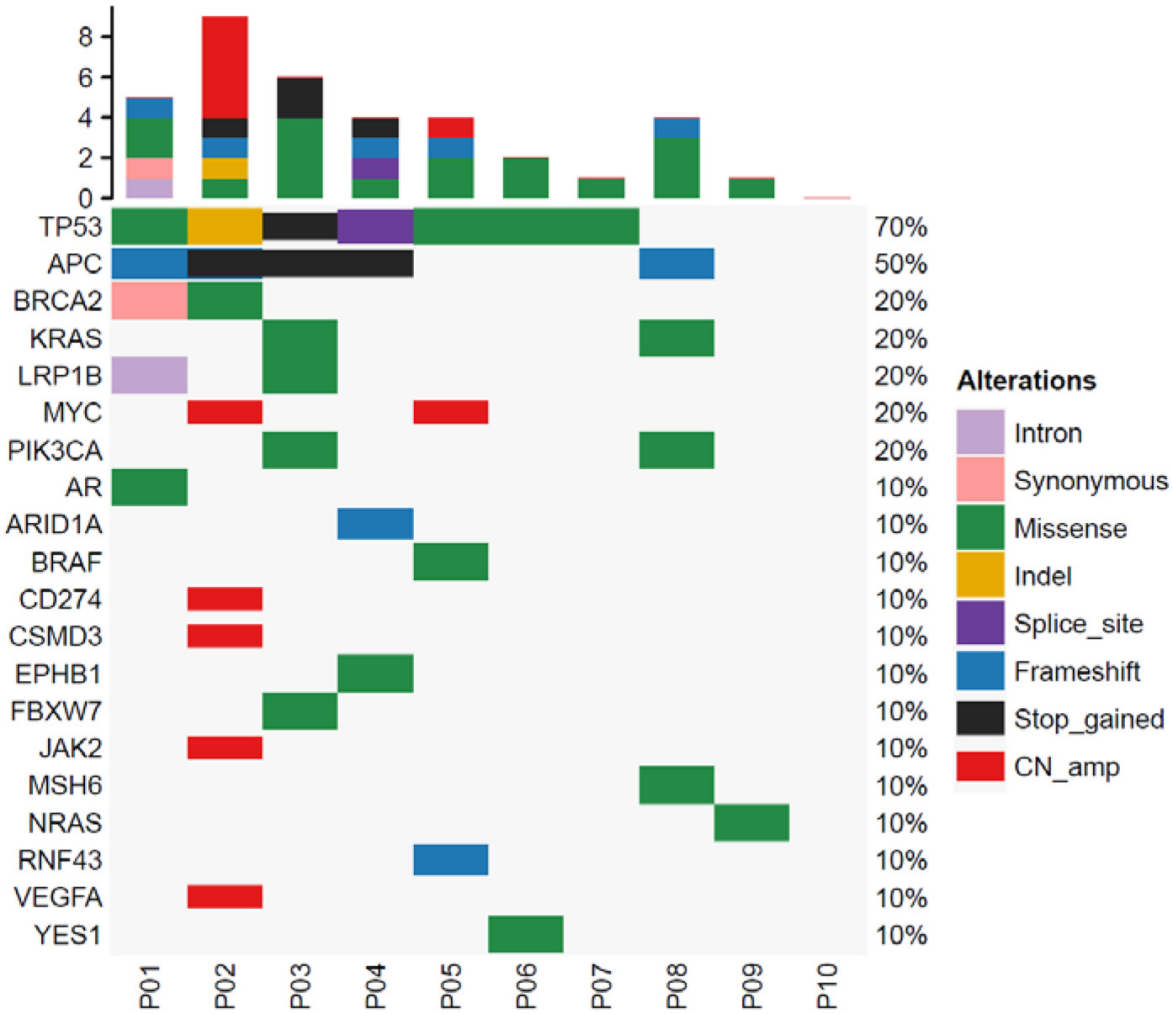
Analysis of cancer-associated gene mutations in colorectal tumors. The top bar plot shows the total number of mutations in cancer-associated genes for each patient. The variant allele frequencies are represented by the right bar. The bottom bar number shows each patient.

The median output per sample by Illumina sequencing was 14,137,745,400 bases, with 47,125,818 sequence reads. The coverage depth was a median 14,409 (IQR 12,812–16,053). Somatic variations detected in cfDNA are shown in Figure 1. The median variation rate per sample was 4, of the 168 cancer-associated genes (IQR 1–4.5 mutations). Variations were identified in the following major cancer-related genes: TP53 (7/10), APC (4/10), BRCA2 (2/10), KRAS (2/10), and LRP1B (2/10). Nine of the ten baseline samples had at least one cancer hotspot variation.

### 3.3 Circulating cell-free DNA dynamics during treatment

**Figure 2:**
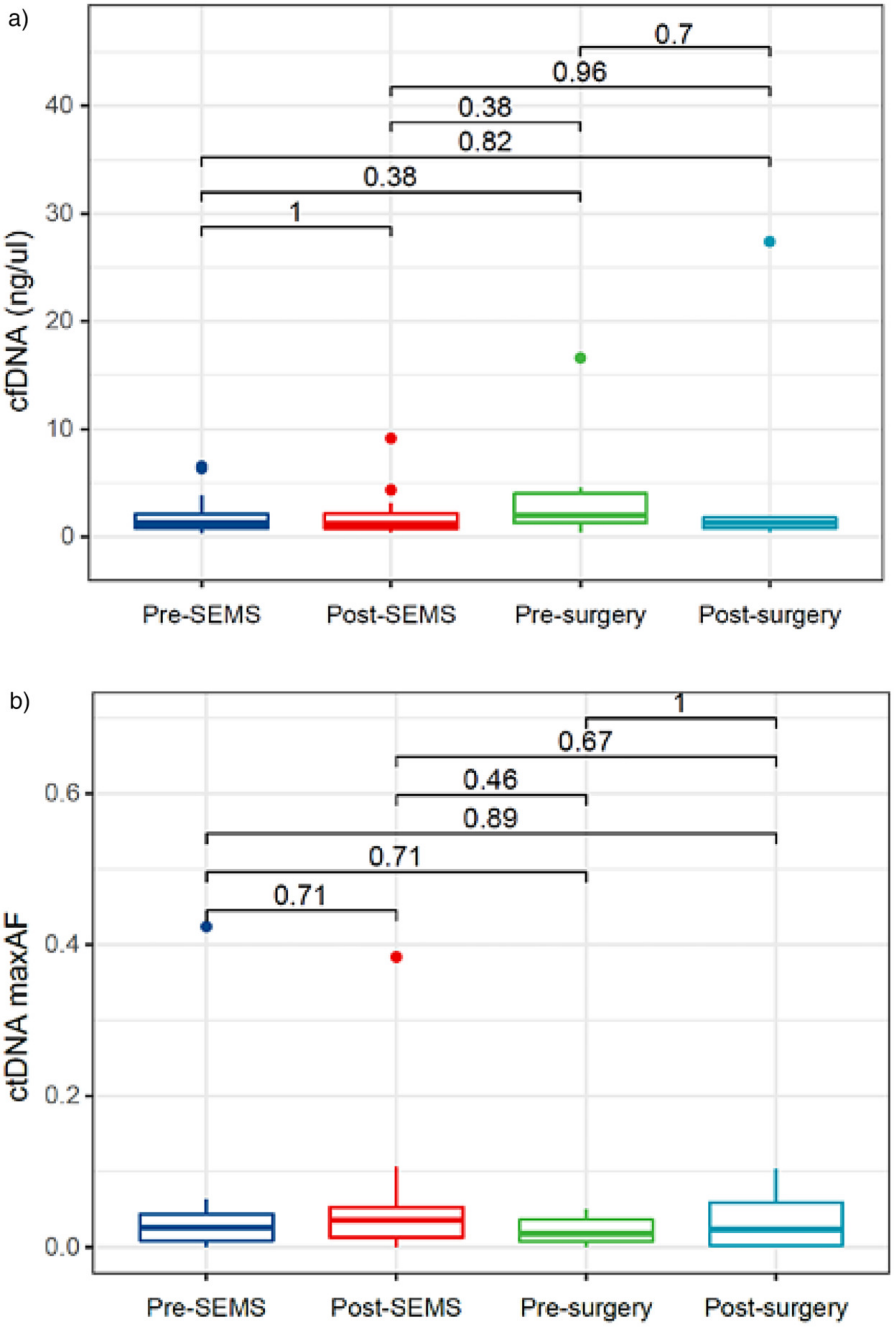
Circulating cell-free DNA and tumor DNA dynamics. **(a)** cfDNA dynamics; **(b)** ctDNA dynamics. cfDNA, circulating cell-free DNA; ctDNA, circulating tumor DNA; SEMS self-expanding metal stent.

Quantitative analyses of cfDNA for 4 time points are shown in Figure 2a. The cfDNA concentration were 1.390 ng/μl at pre-SEMS, 1.195 ng/μl at post-SEMS, 2.310 ng/μl at pre-surgery, 1.399 ng/μl at post-surgery. There were no significant differences between these points.

### 3.4 Circulating tumor DNA dynamics during treatment

Quantitative analyses of ctDNA for 4 time points are shown in Figure 2b. The maxAF represented the somatic mutation detected from the patient, and was defined as the highest allelic fraction observed among all the mutations detected from their blood sample, regardless of gene or mutation site. Despite the reduction in ctDNA maxAF following neoadjuvant chemotherapy, no statistically significant differences were observed across various time points.

### 3.5 Representative circulating tumor changes during treatment

**Figure 3:**
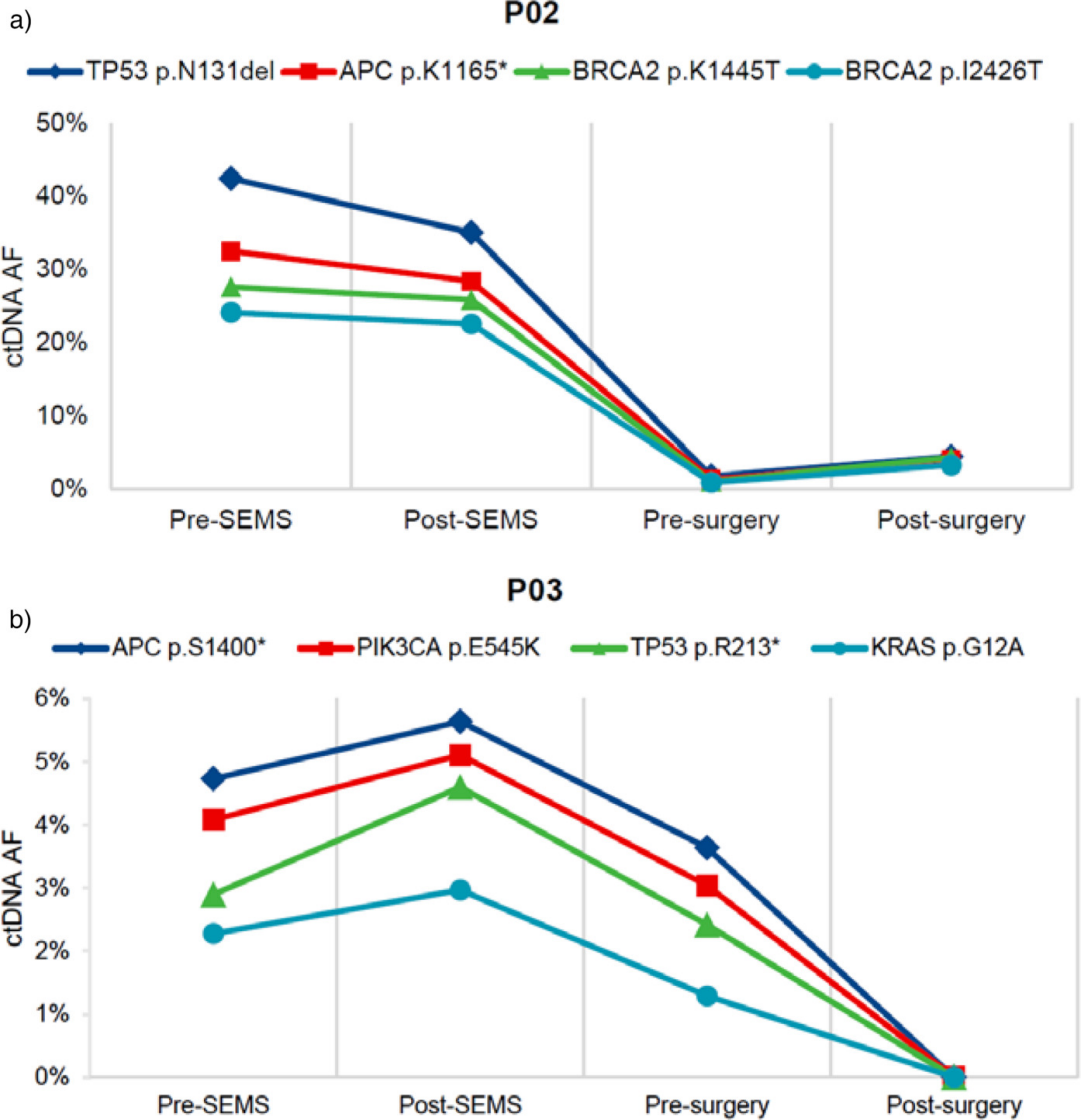
Representative ctDNA changes during treatment. **(a)** The patient presented with sigmoid colon cancer and synchronous liver metastases (case 2). **(b)** The patient presented with descending colon cancer (case 3). TP53, tumor protein p53; APC, adenomatous polyposis coli; BRCA2, breast cancer susceptibility gene 2; PIK3CA, phosphatidylinositol-4,5-bisphosphate 3-kinase catalytic subunit alpha; KRAS, kirsten rat sarcoma viral oncogene homologue; SEMS, self-expanding metal stent; ctDNA, circulating tumor DNA; AF, allele frequency. * nonsense mutation.

We had opportunity to assess changes in ctDNA during the treatment. In case 2, the fraction of mutant fragments of TP53 (p.N131del), APC (p.K1165*), BRCA2 (p.K1445T) and BRCA2 (p.I2426T) decreased sharply after neoadjuvant chemotherapy, but increased slightly at the post-surgery time point (Figure 3a). In case 3, the fraction of mutant fragments of APC (p.S1400*), PIK3CA (p.E545K), TP53 (p.R213*) and KRAS (p.G12A) exhibited a progressive decline from the time point post-SEMS through to the time point post-surgery (Figure 3b).

## 4 Discussion

This study is the first to evaluate the oncological risks of the treatment model combining stent placement (SEMS) with neoadjuvant chemotherapy for obstructive colon cancer by detecting cfDNA and ctDNA levels. Our findings indicate that SEMS does not cause an increase in cfDNA and ctDNA levels, and neoadjuvant treatment does not cause changes in these levels due to delayed surgery, suggesting the safety of this treatment model. Existing studies on stent placement combined with neoadjuvant therapy have reported favorable short-term follow-up outcomes. For instance, Zhang et al. (17) reported a 1-year recurrence-free and metastasis-free rate of 96.8% in patients undergoing this combined treatment, compared to 91.3% in patients undergoing direct surgery. Similarly, Yang et al. (20) reported a 1-year distant metastasis and local recurrence rate of 4.26%.

cfDNA originates from apoptotic or necrotic cells and serves as an important biomarker for cellular damage, while ctDNA is derived from cancer cells undergoing apoptosis or necrosis and may contain cancer-specific gene mutations, such as KRAS (21). As ctDNA levels can reflect tumor burden, it has been used as a personalized biomarker for treatment monitoring (22, 23). The biological half-life of ctDNA is approximately a few hours (24). However, cancer cells experience significant damage following intestinal stent expansion, and previous studies have shown that ctDNA levels can peak 3 days after stent placement (11). In our study, no changes in cfDNA and ctDNA levels were observed following stent placement, and ctDNA levels significantly decreased after neoadjuvant chemotherapy, suggesting the inhibitory effect of chemotherapy on tumors.

ctDNA plays a critical role in early tumor screening, postoperative follow-up, and monitoring treatment effectiveness (24). Dynamic monitoring of ctDNA changes after radical resection of colorectal cancer helps predict the efficacy of postoperative adjuvant chemotherapy and guides further treatment strategies (12, 25). In our Case 2, the patient presented with liver metastases at diagnosis. After stent placement combined with neoadjuvant chemotherapy, ctDNA levels showed a significant decrease. Although surgery removed the primary and metastatic lesions, a slight increase in ctDNA levels was observed postoperatively, indicating the need for additional targeted or other therapies to enhance treatment for such patients.

This study has certain limitations. First, the sample size is small, which may introduce bias and affect statistical outcomes. Second, blood samples were collected only at four time points. Blood sampling during chemotherapy and continuous postoperative monitoring would better reflect the dynamics of ctDNA changes. Third, the relationship between ctDNA and prognosis remains unclear and requires follow-up studies to explore prognostic implications. A larger, randomized trial to compare different groups with different treatment models and prognosis study design would help strengthen the reliability of the conclusions.

## 5 Conclusions

This study evaluated the oncological impact of the treatment model combining stent placement and neoadjuvant chemotherapy by monitoring ctDNA changes. No increase in ctDNA levels was observed after stent placement combined with chemotherapy, suggesting that this treatment model may be a safe and reliable therapy.

## Data Availability

The raw data supporting the conclusions of this article will be made available by the authors, without undue reservation.
